# Percutaneous Ultrasound-Guided Renal Biopsy in Greek Children: 15 Years of Experience at a Single Center

**DOI:** 10.3390/pediatric16040083

**Published:** 2024-11-08

**Authors:** John Dotis, Antonia Kondou, Vasiliki Karava, Pavlos Siolos, Vivian Georgopoulou, George Liapis, Maria Stamou, Fotios Papachristou, Nikoleta Printza

**Affiliations:** 1 Pediatric Nephrology Unit, First Department of Pediatrics, Aristotle University of Thessaloniki, Hippokration General Hospital of Thessaloniki, 54642 Thessaloniki, Greece; tkondou@hotmail.com (A.K.); vasilikikarava@hotmail.fr (V.K.); siolosp@gmail.com (P.S.); nprintza@gmail.com (N.P.); 2 Radiology Department, Hippokration General Hospital of Thessaloniki, 54642 Thessaloniki, Greece; vivian.georgopoulou@gmail.com; 3 First Pathological Anatomy Department, Laikon General Hospital of Athens, 11567 Athens, Greece; pathology@laiko.gr; 4 Second Department of Pediatrics, Aristotle University of Thessaloniki, AHEPA General Hospital of Thessaloniki, 54636 Thessaloniki, Greece; bpedclin@auth.gr

**Keywords:** kidney disease, renal biopsy, histopathology, nephritic/nephrotic syndrome, children

## Abstract

Background: Percutaneous ultrasound-guided renal biopsy (PRB) is a key element for diagnosis and management of several renal pathologies. We aimed to lay out the experience of our pediatric nephrology unit performing PRBs. The rationale and findings of these biopsies, safety issues and considerations of the extracted data are going to be analyzed. Methods: A retrospective study was conducted from 2008 to 2023 based on the review of the medical records of pediatric patients who underwent PRBs. In total, 216 kidney biopsies in 206 patients were performed: 115 (53.2%) during the 2008–2015 period and 101 (46.8%) during the 2016–2023 period. Results: The most frequent clinical indication for PRBs was nephritic syndrome followed by nephrotic syndrome, observed in 84 (40.8%) and 72 (34.9%) patients, respectively. The predominant diagnosis was minimal change disease (MCD) (23.3%), followed by focal segmental glomerulosclerosis (FSGS) (15%) equal to lupus nephritis (LN) (15%), and immunoglobulin A nepropathy (10.2%). Minor complications, such as subcapsular hematomas were observed in approximately 15% of patients while no therapeutic intervention was needed. Conclusions: This report is the first review of pathohistological data covering a pediatric population over a 15-year period in Greece and one of the largest in southeastern Europe, especially in the Balkan region. The main indication for a PRB was nephritic syndrome; however, MCD was the main histological diagnosis. This study emphasis the fact that PRBs constitute a safe and reliable method of diagnostic approach to kidney diseases in childhood and offers important information on therapeutic approaches as well as the prognosis of these patients.

## 1. Introduction

Chronic kidney disease (CKD) in the pediatric population is a major public health problem with a rising prevalence and considerable implications for the patients and the healthcare system [1]. Among the vast etiologies of CKD, congenital genitourinary abnormalities and glomerular diseases account for the majority of cases in children, in contrast to the adult population [2]. Although the standard workup of structural abnormalities is laboratory in combination with imaging studies, many kidney diseases can only be diagnosed though biopsy. In fact, many treatment protocols have renal biopsy as a prerequisite [3,4]. Furthermore, the classification of many kidney diseases is merely histological, and histology determines prognosis and treatment response. Regarding CKD, it is estimated that 22.3% of cases in children are due to glomerular diseases [4]. Renal biopsy with the use of modern stains and immunofluorescence has revolutionized the understanding of both glomerular and tubular disorders, offering novel and targeted therapies [5]. Percutaneous ultrasound-guided renal biopsy (PRB) is a minimally invasive procedure and despite its technical challenges, such as possible lack of cooperation and size variation in children, it is considered safe [3,6]. Although there is no unanimous agreement on when a patient should undergo a biopsy, commonly accepted indications exist and are individualized in each case. Epidemiologic data from other pediatric centers are in accordance with that of our own and most patients are biopsied to further evaluate idiopathic nephrotic syndrome (NS), especially steroid-resistant (SRNS) and steroid-dependent (SDNS) subtypes [4,7]. Other common indications include significant proteinuria (>0.5 g/g creatinine in urine) accompanied with hypertension or elevated serum creatinine, combined hematuria and proteinuria in the presence of red flags such as normal C3 levels, acute kidney injury (AKI) that is not improving, hypertension, or family history of CKD (4). Other absolute indications for renal biopsy include patients with a high suspicion of rapidly progressive glomerulonephritis (RPGN) [8] and patients with systemic lupus erythematosus (SLE) and renal involvement to further classify the lupus nephritis (LN) [9].

Reported biopsy complications usually result from and are dependent on a variety of biopsy techniques and instruments. Most common are clinically less relevant events like microscopic hematuria, transient gross hematuria, and asymptomatic minor subcapsular hematoma [10]. Nowadays, major complications are extremely rare, including either puncture damage to nearby organs or infections or gross hematuria which need a blood transfusion and/or surgery or catheter-based intervention (embolization) secondary to bleeding [10,11].

The purpose of this study is to lay out the 15-year experience of the sole tertiary pediatric nephrology unit of northern Greece performing renal biopsies. The rationale and findings of these biopsies, safety issues and considerations of the extracted data are going to be analyzed.

## 2. Materials and Methods

### 2.1. Participants

A retrospective study was conducted from 1 July 2008 to 30 June 2023 at the Pediatric Nephrology Unit of our department, based on the review of the medical records of pediatric patients who underwent PRB. For all patients, demographic data including age, gender and ethnicity, clinical and laboratory findings, indication for biopsy, result of the histopathological examination, as well as any complications were recorded.

### 2.2. Diagnostic Criteria

KDIGO 2021 guidelines were followed in developing the diagnostic criteria for renal biopsy in this study [12]. Corticosteroid-resistant nephrotic syndrome or nephritic syndrome with extensive hematuria, severe proteinuria, decreased kidney function, and a low level of C3 that persists for more than 2 months were all regarded as inclusion criteria that a kidney biopsy was necessary. In addition, patients with atypical age including <12 months and >10 years at presentation, as well as patients with persistent hematuria with proteinuria underwent kidney biopsy. In cases where parents did not provide consent or in patients with bleeding diathesis and coagulation abnormalities, uncontrolled hypertension, acute pyelonephritis, single kidney, and severe anemia were among the exclusion criteria from the study.

Inadequate or non-diagnostic biopsy was defined with any of the following terms: “inadequate for diagnosis”, “insufficient for diagnosis”, only “medulla” and/or “fat” and/or “connective tissue” and/or “skeletal muscle” and/or <7 glomeruli, were found on histopathological results.

### 2.3. Procedure

All biopsies were performed using either 16-gauge or 18-gauge biopsy needles. In each biopsy, two kidney tissue samples were taken, while, in certain conditions three samples were received (electronic microscopy necessity). From 2008–2010, manual needles were used and afterwards were replaced with semi-automatic biopsy needles. In all patients, before performing the biopsy procedure, a pre-operative test (complete blood count test, coagulation mechanism check) and renal function tests were obtained. Real-time ultrasound guidance was used for all biopsies, with a pediatric nephrologist being assisted by an ultrasonographer. All kidney tissue sections were examined with common microscopes as well as immunofluorescence analysis, periodic acid-Schiff (PAS), and hematoxylin and eosin (H&E) staining. Patients follow a certain post-biopsy protocol. Specifically, all renal biopsies in our study were performed in an in-hospital setting and patients were ordered to strict bed rest for about 24 h following the procedure for their safety because major complications may occur up to the first 24 h. After the biopsy, patients lie down for 4–6 h without getting out of bed. A close monitoring of blood pressure, pulse, and breathing in addition to a blood count test, urinalysis and a renal ultrasonography the same and the following day were performed. In addition, the patients were advised to avoid vigorous activity for approximately 2 weeks after being discharged from the hospital.

It is noted that renal graft biopsies were not included in the study. Before the biopsy procedure, the parents or guardians were informed in detail and written consent was requested. Retrospective chart reviews of existing medical records, such as our study, do not require a prospective Institutional Review Board (IRB) statement approval because the intent is to obtain clinical information for teaching purposes.

### 2.4. Plan of Statistical Analyses

The amount of data divided into two equal time periods based on total study interval. This was considered appropriate in order to investigate whether there were significant changes in results during progression over the years. One of the main advantages of period-to-period comparison is the ability to identify trends and patterns over the course of the study. Statistical analysis of the data was conducted using IBM SPSS Statistics for Windows, Version 22.0 (IBM Corp., Armonk, NY, USA). Descriptive statistics such as percentages, means, and medians were calculated. The Kolmogorov–Smirnov test was used to determine the normality of data distribution. Differences in the frequency of various glomerular diseases among two study periods were compared using the chi-square test and Fisher exact test. In addition, *p* < 0.05 was considered statistically significant.

## 3. Results

In total, 216 renal biopsies in 206 patients were performed: 115 (53.2%) during the 2008–2015 period and 101 (46.8%) during the 2016–2023 period. Ten patients underwent a second renal biopsy. The most frequent diagnosis at first renal biopsy were minimal change disease (MCD), focal segmental glomerulosclerosis (FSGS), LN and immunoglobulin A nephropathy (IgAN), detected in 48 (23.3%), 31 (15%), 31 (15%) and 21 (10.2%) patients, respectively, followed by other rare pathologies (Table 1).

**Figure 1:**
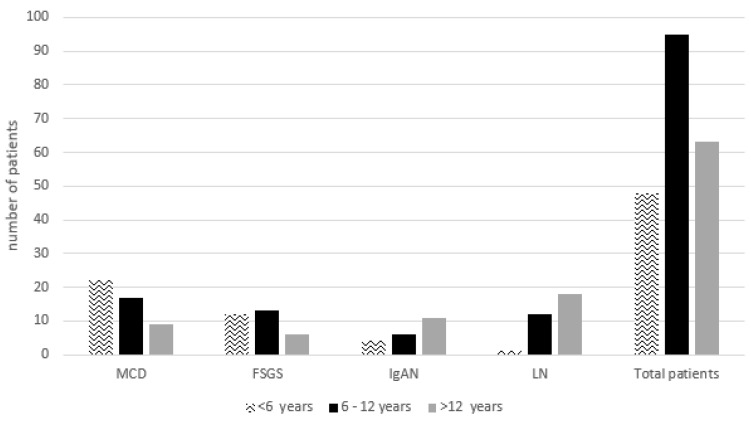
Differences of renal biopsy diagnosis based on age group. MCD minimal change disease, FSGS focal segmental glomerulosclerosis, IgAN immunoglobulin A nephropathy, LN lupus nephritis.

Of note, renal biopsy was non-diagnostic in 25 (12.1%) patients. Moreover, the prevalence of non-diagnostic renal biopsy decreased from 15% during the 2008–2015 period to 8.6% during the 2016–2023 period. The prevalence of membranous nephropathy (MN) and immunoglobulin A vasculitis (IgAV) increased, from 1.8% and 3.5% during the 2008–2015 period to 7.5% and 8.6% during the 2016–2023 period, respectively. Based on age, the most common finding after the first renal biopsy was MCD in age groups <6 years (45.8%) and 6–12 years (17.9%). However, in age group of >12 years LN (28.6%) was the predominant diagnosis (Figure 1).

The most frequent clinical indication for first renal biopsy was nephritic syndrome in 84 (40.8%) patients closely followed by NS observed in 72 (34.9%) patients. The prevalence of first renal biopsy for isolated hematuria and CKD decreased from 8% and 3.5% during the 2008–2015 period to 4.3% and 1.1% during the 2016–2023 period, respectively.

Among the 84 patients with nephritic syndrome, nephrotic range proteinuria was noted in 11 (13.1%) and AKI in 7 (8.3%) patients. LN was observed in 20 (23.8%) and IgAN in 17 (20.2%) patients, which were the two most common renal biopsy findings, followed by IgAV in 12 (14.3%) and post-infectious glomerulonephritis (PIGN) in 10 (11.9%) patients. Of note, based on the renal biopsy results, no diagnosis was made in 10 (11.9%) patients (Table 2).

The age at diagnosis among the 4 more frequent kidney diseases significantly differ (*p* < 0.001), with a highest age observed in patients with LN (median age: 13 years, range 7–15.5) and the lowest in patients with IgAV (median age: 7 years, range 2.5–11). The prevalence of IgAV increased from 9.1% to 20%, while the prevalence of PIGN decreased from 15.9% to 7.5% between the two on-study periods. Of note, 5 patients out of 10 underwent a second renal biopsy within a median period of 2 years (range 1–5); one patient with antineutrophilic cytoplasmic antibody-associated vasculitis (AAV), one with MCD, one with LN and two with non-diagnostic renal biopsy. For the other 5 patients that underwent second renal biopsy within a median period of 8.5 years (range 6–13), diagnosis of Alport syndrome, based on electronic microscopy findings and IgM nephropathy were made in 2 patients with initial non-diagnostic kidney biopsies. The second renal biopsy of a patient with AAV, due to first biopsy findings, revealed IgAV. In one patient with an initial C1q nephropathy (C1qN) that underwent a second renal biopsy 7 years later, the presence of FSGS was revealed. Finally, the second biopsy result of MCD did not change in the remaining patient.

**Figure 2:**
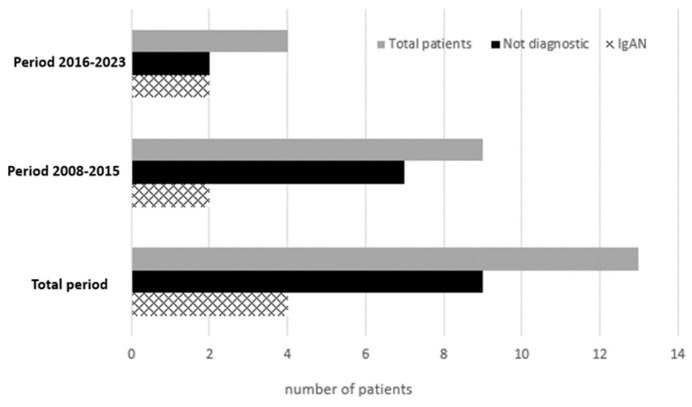
Distribution of renal biopsy diagnoses between the two study periods in patients presented with isolated hematuria. IgAN immunoglobulin A nephropathy.

Among the 13 patients with isolated hematuria, non-diagnostic renal biopsy was observed in 9 (69.2%) patients and IgAN in the other 4 (30.8%) (Figure 2). The prevalence of non-diagnostic renal biopsy showed a decreasing trend between the two on-study periods from 77.8% to 50%. Of note, among the 9 patients with non-diagnostic renal biopsy, Alport syndrome was revealed at the genetic analysis of 2 patients out of 6 that had conducted the analysis. None of the patients with isolated hematuria underwent a second renal biopsy during the study period.

Among the 32 patients presented with proteinuria without hematuria, AKI was found in 7 (22.6%) patients. LN and tubulointerstitial nephritis (TIN) were the most frequent renal biopsy results, observed in 11 (34.4%) and 8 (25%) patients, respectively (Table 3).

The renal biopsy was non-diagnostic in 6 (18.8%) patients. Of note, among the patients with TIN, two patients presented tubulointerstitial nephritis uveitis (TINU) syndrome. Lastly, renal biopsy for CKD of unknown origin was performed in 5 patients, which revealed the presence of chronic TIN.

Regarding complications, there were no serious complications in our center, while small subcapsular hematomas were observed in approximately 15% of patients. No therapeutic intervention was required in any case, while all of them were fully absorbed within 5 to 10 days.

## 4. Discussion

The present study provides data on both the indications for renal biopsy in children and the most common etiology of kidney disease based on histopathological findings and confirms the safety and efficacy of renal biopsy in children for an extensive follow-up period. To the best of our knowledge, based on the literature, the number of 216 biopsies in pediatric patients constitute the largest number with such data coming from Greece and one of the largest in southeastern Europe, especially in the Balkan region. In addition, the failure rate of our center was 12.1%, which is not significantly different from those reported in the relevant literature, while no serious complications were observed due to the rigorous follow-up protocol being followed [13,14].

There was a reduction in the number of biopsies from the first to the second period. On one hand, this was probably the result of the strictest criteria established for renal biopsy through the years to reduce complications and on the other hand, the rise of genetic testing in pediatric nephrology. In addition, despite the safety of biopsies by experienced healthcare practitioners the procedure, due to several potential risks, should be reserved for certain patients in which empirical treatment is not the optimal option.

Nephritic syndrome was the commonest indication for renal biopsy in our center while NS was the second. Regarding nephritic syndrome, LN, IgAN, IgAV, PIGN were the most frequent histopathological diagnoses. These findings are quite similar to reports originating from China, especially [2,15]. As described in the results, there was a difference in the age group for each diagnosis; however, it is worth mentioning that the prevalence of IGAN remained stable between the two time periods studied, while IgAN and IgAV showed an upward trend and PIGN showed a downward shift. The decrease in PIGN probably reflects the fact that nowadays PIGN is not an indication for biopsy as the diagnosis can be confirmed by clinical and laboratory findings. In addition, the widespread administration of antibiotics and the often-benign nature of the disease rarely require renal biopsy, except when other glomerular pathologies are suspected [16]. Both IgAN and IgAV prevalence increased which could be attributed to the need for targeted and more aggressive treatment early in the course of the disease [17].

LN consistently remains the most frequent histological finding in children aged more than 12 years in our study. In the majority of cases, LN appears as a nephritic syndrome, while in 1/3 of them, isolated proteinuria is the indication for renal biopsy. It has been established that LN due to SLE occurring before 18 years of age, accounts for 10–20% of cases and is known to have a more aggressive onset compared to in adults [18]. Specifically, childhood-onset SLE manifests a more aggressive disease course and higher disease activity than adult-onset SLE, leading to morbidity, mortality, and irreversible damage to major organs, commonly including the kidneys [18].

In our study, MCD was the most common pathology for NS cases and also for total cases (23.3% overall). In addition, we found a marginal increase from 22.1% to 24.7% between the two study periods. This is in accordance with multiple studies reporting MCD as the most common histopathology in pediatric biopsies [19,20,21,22]. However, Czech Registry of Renal Biopsies from the period between 1994 and 2011, showed that MCD was the most frequent finding in children with nephrotic proteinuria approaching 39.7% [19]. Ιn two comparative studies from the region of southern Croatia, the frequency of MCD increased from 4.6 to 16.7% between 1995 and 2005 vs. 2008 and 2017, respectively [20,21]. Although these numbers show a significantly increasing trend, they are still relatively smaller than in Czech studies and are about the same as the Norwegian Kidney Biopsy Registry in the which frequency of MCD was 16% [22], comparable to the findings of our study. Instinctually, the following question arises: Is there a need for a biopsy to diagnose MCD? Unfortunately, there is currently no noninvasive procedure or practical classification method for distinguishing MCD from other primary glomerular diseases available and in cases indicating a renal biopsy should be performed.

Of note, MN frequency has increased between the two on-study periods, mainly in children with NS. Specifically for MN, in a study from China there was an increase in proportion from 3% between the years 2004 and 2007 to 7% between the years 2012 and 2014, which are similar to our findings (1.8% and 7.5% between the years 2008 and 2015 and 2016 and 2023, respectively) [2]. Coming back to MCD, it was found in our study that it remains the most common histopathological diagnosis in children aged less than 12 years followed by FSGS which is the second most common in the same age group. It should be noted that the rather high percentage of FSGS in our patients is probably due to the criteria followed in our center for performing renal biopsy. SRNS is an absolute indication for renal biopsy in contrast to steroid sensitive NS (SSNS) where renal biopsy is performed under conditions such as frequent recurrences or SDNS [22].

The majority of the reported complications of renal biopsies concern vasculature. Macroscopic hematuria has been reported at a rate of 5–20% while microscopic hematuria is present postoperatively in virtually all patients [3]. Perinephric hematoma is another common complication affecting up to 18% of patients [10]. In our study, small subcapsular hematomas were the most common adverse event observed in approximately 15% of patients, probably because of a more systematic use of routine ultrasonographic examination after the biopsy (same day and next day), and this finding must be interpreted with caution. However, no therapeutic intervention was required, while the hematomas were absorbed within a few days. A relatively rare complication reported in the literature is the formation of arteriovenous fistula requiring invasive interventions such as endovascular embolization [11]. More severe complications such as the puncturing of other organs, kidney loss, infections, or even death have been described but their incidence is so low that the benefits outweigh the risks in patients with no contraindications to undergo the procedure [11,13,23]. However, the lack of globally agreed standards of practice leaves room for possible overuse or procedure-related errors [6]. Nowadays, the development of more sophisticated and less invasive diagnostic tools such as genetic testing for Alport syndrome or congenital NS raises a question of superiority and efficacy of the PRB [24,25].

## 5. Limitations

Although this study provides numerus data for the estimation of trends in the frequency of diagnosing renal biopsy glomerular disease in Greek children and includes an extensive follow-up period, it has several limitations that should be noted. First, this was a single-center study although it reflects approximately 1/3 of Greece population based on the area and population coverage of our unit. Second, since we reported the biopsy results as proportions, unfortunately, we could not accurately report the incidence rates of the different histologic subtypes. Additionally, our results may be skewed because we only included cases from northern Greece. Therefore, our conclusions may not be applicable to other Greek regions.

## 6. Conclusions

In this pediatric population studied in northern Greece, nephritic syndrome constitutes the most common indication for renal biopsy, followed very closely by NS. The most frequently found pathology was MCD, while it was interesting to note that an increased proportion of LN was revealed. We found a considerable shift in the frequency of many glomerular disease subtypes between the two time periods studied and we will we follow their evolution in the coming years. Finally, PRBs constitute a safe and reliable method of diagnostic approach to pediatric kidney diseases and must be used under certain circumstances and indications. Under these conditions, important information on therapeutics approach as well as the prognosis of these patients is offered. The present data are an important contribution to the epidemiology of renal diseases in southeastern Europe and add our knowledge gained from a few previous studies on this region.

## Figures and Tables

**Table 1:** Distribution of renal biopsy diagnoses between the two study periods.

Diagnosis	Total Period (n)	%	2008–2015 (n)	%	2016–2023 (n)	%	*p*
MCD	48	23.3	25	22.1	23	24.7	0.741
FSGS	31	15.0	20	17.7	11	11.8	0.327
LN	31	15.0	18	15.9	13	14.0	0.845
IgAN	21	10.2	9	8.0	12	12.9	0.258
TIN	13	6.3	7	6.2	6	6.5	>0.999
IgAV	12	5.8	4	3.5	8	8.6	0.144
PIGN	10	4.9	7	6.2	3	3.2	0.517
MN	9	4.4	2	1.8	7	7.5	0.08
AAV	3	1.5	1	0.9	2	2.2	0.590
ATN	1	0.5	1	0.9	0		
C1qN	1	0.5	1	0.9	0		
IgMN	1	0.5	1	0.9	0		
Not diagnostic	25	12.1	17	15.0	8	8.6	0.199
Total patients	206	100	113	100	93	100	

MCD minimal change disease, FSGS focal segmental glomerulosclerosis, LN lupus nephritis, IgAN immunoglobulin A nephropathy, TIN tubulointerstitial nephritis, IgAV immunoglobulin A vasculitis, PIGN post-infectious glomerulonephritis, MN membranous nephropathy, AAV antineutrophilic cytoplasmic antibody-associated vasculitis, ATN acute tubular necrosis, C1qN C1q nephropathy, IgMN immunoglobulin M nephropathy.

**Table 2:** Distribution of renal biopsy diagnoses between the two study periods in patients presented with nephritic syndrome.

Diagnosis	Total Period (n)	%	2008–2015 (n)	%	2016–2023 (n)	%	*p*
LN	20	23.8	12	27.3	8	20	0.456
IgAN	17	20.2	7	15.9	10	25	0.416
IgAV	12	14.3	4	9.1	8	20	0.215
PIGN	10	11.9	7	15.9	3	7.5	0.312
FSGS	7	8.3	5	11.3	2	5	0.427
AAV	3	3.6	1	2.3	2	5	0.603
MCD	3	3.6	1	2.3	2	5	0.603
MN	2	2.4	0	0	2	5	
Not diagnostic	10	11.9	7	15.9	3	7.5	0.312
Total patients(n)	84	100	44	100	40	100	

LN lupus nephritis, IgAN immunoglobulin A nephropathy, IgAV immunoglobulin A vasculitis, PIGN post-infectious glomerulonephritis, FSGS focal segmental glomerulosclerosis, AAV antineutrophilic cytoplasmic antibody-associated vasculitis, MCD minimal change disease MN membranous nephropathy.

**Table 3:** Distribution of renal biopsy diagnoses between the two study periods in patients presented with proteinuria without hematuria.

Diagnosis	Total Period (n)	%	2008–2015 (n)	%	2016–2023 (n)	100%	*p*
LN	11	34.4	6	35.3	5	33.3	>0.999
TIN	8	25.0	3	17.6	5	33.3	0.423
FSGS	2	6.3	0	0	2	13.3	
ATN	1	3.1	1	5.9	0	0	
C1q nephropathy	1	3.1	1	5.9	0	0	
MCD	1	3.1	1	5.9	0	0	
MN	2	6.3	2	11.8	0	0	
Not diagnostic	6	18.8	3	17.6	3	20	>0.999
Total patients	32	100	17	100	15	100	

LN lupus nephritis, TIN tubulointerstitial nephritis, FSGS focal segmental glomerulosclerosis, ATN acute tubular necrosis, C1qN C1q nephropathy, MCD minimal change disease, MN membranous nephropathy.

## Data Availability

Data are available upon request.
